# Evaluation of Pressing Issues in Ecological Momentary Assessment

**DOI:** 10.1146/annurev-clinpsy-080921-083128

**Published:** 2022-12-07

**Authors:** Arthur A. Stone, Stefan Schneider, Joshua M. Smyth

**Affiliations:** 1 Department of Psychology, University of Southern California, Los Angeles, California, USA; 2 Department of Biobehavioral Health, Pennsylvania State University, University Park, Pennsylvania, USA

**Keywords:** Ecological Momentary Assessment, Experience Sampling Method, content validity, gold standard, training, missingness, reliability, selection bias, comparison standards

## Abstract

The use of repeated, momentary, real-world assessment methods known as the Experience Sampling Method and Ecological Momentary Assessment (EMA) has been broadly embraced over the last few decades. These methods have extended our assessment reach beyond lengthy retrospective self-reports as they can capture everyday experiences in their immediate context, including affect, behavior, symptoms, and cognitions. In this review we evaluate nine conceptual, methodological, and psychometric issues about EMA with the goal of stimulating conversation and guiding future research on these matters: the extent to which participants are actually reporting momentary experiences, respondents’ interpretation of momentary questions, the use of comparison standards in responding, efforts to increase the EMA reporting period beyond the moment to longer periods within a day, training of EMA study participants, concerns about selection bias of respondents, the impact of missing EMA assessments, the reliability of momentary data, and for which purposes EMA might be considered a gold standard for assessment. Resolution of these issues should have far-reaching implications for advancing the field.

## INTRODUCTION

Behavioral science research has resulted in scores of psychometrically sound self-report instruments about thoughts, opinions, feelings, events, and behaviors that are intended to be summaries of significant time periods (retrospective reports) or to characterize a person’s usual levels and dispositions across situations or over time (trait or global reports). In light of some limitations of these traditional assessment options, researchers have long wished for methods to collect higher-resolution data with greater ecological validity (Brunswik 1941). Higher resolution is desired so that associations between immediate contexts and experiences can be examined and so that dynamic processes occurring over relatively short time periods (minutes, hours, days) can be explored; greater ecological validity is desired so that observed associations and processes are representative of respondents’ everyday lives.

These aspirations have yielded a collection of within-day momentary data capture methodologies. Selected examples of, and reviews about, momentary research are presented in papers by the following authors: Bolger et al. (2003), Conner & Barrett (2012), Csikszentmihalyi & Hunter (2003), Degroote et al. (2020), DeVries (1987), Ebner-Priemer & Trull (2009), Fisher & To (2012), Gorin & Stone (2001), Hamaker & Wichers (2017), Heron et al. (2017), Kirtley et al. (2021), Myin-Germeys et al. (2009), Reis & Gable (2000), Scollon et al. (2009), Shiffman et al. (2008), Smyth & Stone (2003), Smyth et al. (2017), Stone & Broderick (2007), Stone & Shiffman (1994), Stone et al. (2007, 2021), and Trull & Ebner-Priemer (2020). Several sources provide summaries and discussion of the analytic techniques used for these complex data (Bolger & Laurenceau 2013, Mehl & Conner 2011, Schwartz & Stone 2007, Shiffman 2014). A core feature of Ecological Momentary Assessment (EMA) methods is the short duration of reporting periods; this is meant to reduce bias and error attributable to inherent memory limitations and to limit the use of cognitive heuristics in self-reports. Relatively unobtrusive in-the-field data collection as respondents go through their everyday lives is intended to ensure ecological validity. Following a first generation of paper-and-pencil methodologies (DeVries 1987), advances in smartphone, Internet, and computer-assisted applications have further increased the appeal of within-day assessments, opening new doors for studying behavior, affect, cognition, and their associations in everyday life.

In this review we focus on the granular, momentary, self-report data collection methods known as the Experience Sampling Method and Ecological Momentary Assessment; we refer to these methods collectively as EMA throughout this review. Developed in the early 1970s, the methods signal respondents at deliberately selected points over time (fixed, random, and/or event-contingent), and respondents answer a brief set of questions tapping the constructs under investigation (Shiffman et al. 2008). Affective states, pain, symptoms, consumption behaviors, social interactions, physical activity, location, stressful events, and dozens of other variables have been the focus of EMA studies. Given the wide applicability and strengths of these methods, it should not be surprising that EMA has been widely adopted: To date, over 1,700 publications have employed momentary data collection in behavioral science and health care. In this review we discuss several issues about EMA methods that we and other researchers have identified over the years while conducting momentary research.

Of the many issues that could be discussed, we have selected nine that we believe are particularly timely for advancing the science of EMA. We raise these concerns with an open mind; we do not know the degree to which the issues seriously threaten the interpretation of EMA data. To be clear, we are not saying that EMA methods are seriously flawed or yield flawed data. On the contrary, we suggest that sober evaluation of these topics, and perhaps remediation of some of them, will increase the utility of EMA. We also do not claim to have solutions for these concerns, but we have suggested avenues of research that may merit consideration. We begin with several issues about the fidelity of the methods as currently implemented in EMA including whether participants are actually reporting momentary experiences, respondents’ interpretation of momentary questions and the use of comparison standards in answering EMA questions, the development of EMA methods with longer recall periods, and the importance of training participants to properly complete EMA assessments. We then move to topics about the data resulting from EMA studies and their interpretation including subject selection bias, moment selection bias and missing data, and the reliability of EMA measures. The review concludes with a discussion about when EMA measures could be considered assessment gold standards.

Most of the topics we discuss have received little research attention, perhaps because researchers believe that momentary reports generally avoid sources of error and bias and that there is therefore no need to examine methodological issues and the psychometric properties of the resulting data. Another possible reason is the difficulty of addressing these issues; for example, many traditional psychometric approaches are not readily applicable to EMA data and processes. Yet another possibility is that the absence of an objective standard for many EMA-related targets (e.g., intrapsychic states) impedes efforts to validate momentary methodologies.

## ARE PARTICIPANTS REPORTING THEIR MOMENTARY EXPERIENCES?

A core assumption underlying the methodology of EMA is that participants who are asked to report their experience at the moment before the prompt actually follow this instruction. It is assumed that EMA reports do not include information about other time periods (e.g., the last day), about a person’s general beliefs, or about judgments that are based upon additional reflection on the momentary experience. EMA procedures that do not measure information about the specified time period have the potential to undermine our understanding of the phenomena they purport to measure and to distort their associations with other variables. If, for example, an EMA respondent was requested to report their affect at a targeted moment but instead reported their “typical” affective state, then we would learn little about actual levels of immediate mood, its variability over time and temporal patterns, or its associations with other time-varying phenomena. And of considerable gravity is the fact that the construct validity of “immediate affect” would be tainted, with all of the resulting consequences for theory testing. Herein lies the importance of this issue.

Conceptual arguments can be both supporting and challenging to the assumption that EMA respondents report their momentary experiences as intended. The first perspective is the accessibility model of emotion articulated by Robinson & Clore (2002) that is often cited in support of using momentary reporting (e.g., Conner & Barrett 2012) to avoid recall bias. The model posits that when people have low accessibility to information in memory, they tend to use semantic memory or beliefs in formulating their recollection. Therefore, self-reports with relatively long recall periods and low accessibility will tend to be based on beliefs and implicit theories (Ross 1989). Conversely, for somewhat shorter recall periods, such as self-reports of the past day, selected details of the experiences tied to specific times and contexts are still accessible from episodic memory, but people may need to use mental shortcuts (cognitive heuristics) to fill in memory gaps to summarize their experiences (Kahneman 2011). In contrast, momentary experiences should be readily accessible from working memory.

The second perspective acknowledges that simple tasks pertaining to answering questions, including momentary ones, often go awry. Theories of survey responses (Tourangeau et al. 2000) argue that respondents sometimes misinterpret even well-formulated questions and that participants may “help” researchers by providing the answers they believe are being sought by the question (Schwarz 1999). Participants often rely on implicit assumptions governing regular conversations in everyday life (Schwarz 1999). Communicating how one felt “just” before being signaled, for instance, is not necessarily most relevant in the context of an ongoing conversation. So, a momentary assessment of anger might ask, “How angry were you just before the signal?” but a participant not experiencing much momentary anger might instead base their answer on a social interaction an hour or two ago that produced the emotion. Such well-intentioned attempts to convey the most relevant information might be sensible in regular conversation, but they do not yield momentary data.

A third perspective that seems to have received less attention recognizes the intrinsic difficulty of collecting information about people’s momentary experiences. Hurlburt and colleagues (Heavey et al. 2012, Hurlburt 1997, Hurlburt et al. 2017) have spent many years working on techniques for the assessment of “pristine inner experience,” defined as momentary reports that are “unspoiled by the act of observation or reflection” (Hurlburt & Akhter 2006, p. 272). The main concern is that participants may report their judgments about experiences rather than providing information about the experiences in their natural states (thus distorting the information). Hurlburt and colleagues have developed the complex and lengthy Descriptive Experience Sampling interview, which comprises procedures intended to overcome this challenge. Although EMA protocols may train participants before letting them loose in the field, Hurlburt and colleagues’ writings (Hurlburt & Heavey 2015) challenge the assumption that EMA procedures accurately capture information about experience prior to a prompt.

We therefore think it is prudent to investigate whether EMA techniques in fact elicit purely momentary information. Might individuals answer EMA questions in a way that is conflated with extraneous information? Given the concerns about what respondents are reporting, we suggest that it is essential to understand how momentary questions are answered. One possibility is to draw on techniques of cognitive interviewing (Willis 2005), a standard tool for the development of survey questions. Cognitive interviewing methods such as instructing participants to describe their thoughts as they answer questions (stream of thought) are akin to the techniques of Hurlburt, albeit with much less probing. We recently conducted a small-scale study to evaluate this issue using brief telephone interviews that incorporated cognitive interviewing techniques as participants went through a typical day (real-time cognitive interviewing) (Wen et al. 2021). Similar to EMA, the interviewing occurred in an everyday environment, and assessments were scheduled at random intervals. At each assessment, we asked respondents which time period they considered when rating their momentary experience (e.g., their anxiety level). We found that, with minimal training about how to complete the momentary ratings, 68% of participants explicitly reported that they focused on the moment before the call. In contrast, with more extensive training, 98% reported using the correct time frame (for more detailed discussion of EMA training, see the section titled How Important Is Training Participants for EMA?). We recognize potential shortcomings of cognitive interviewing for addressing the issue under discussion, but contend that these data provide some support that current momentary methodologies are effective at capturing the desired reporting period (particularly when coupled with good training).

Other approaches to investigate reporting processes are possible, including experimental paradigms wherein participants’ moods, for example, are manipulated throughout a period of time so that the researcher objectively knows the stimuli underlying what was experienced at a given moment in the experiment. Participants could be prompted at selected times to report their momentary moods, which could be compared with the concurrently elicited emotion. Relatively strong correspondence would indicate that momentary reports were measuring as intended, whereas poor correspondence would suggest that they were not. We acknowledge that various processes such as the level of attention to the stimuli and the potency of the manipulations could undermine the validity of such experiments. Nevertheless, we recommend that studies like these be conducted to further support the validity of momentary data capture techniques.

## HOW DO RESPONDENTS INTERPRET THE CONTENT OF EMA QUESTIONS?

In this section we consider how participants interpret the content presented in EMA questions and, particularly, if they are interpreting them as we expect them to. EMA questions are rarely developed using rigorous and systematic pilot testing of content validity, such as focus groups and cognitive interviews to evaluate how they are understood by respondents—a point emphasized in a review on EMA in physical activity studies (Degroote et al. 2020) (for an exception, see Boesen et al. 2018). Instead, questions employed in EMA protocols are often created based on their face validity in the eyes of the investigators, or they are derived and modified from questionnaires that encompass much longer time frames (Boesen et al. 2018, Degroote et al. 2020).

It is plausible that the meanings of ostensibly clear-cut questions such as “How angry were you?” are altered when questions are converted from a reference period spanning several days or weeks to the immediate moment before an EMA prompt or to a brief time period just before the prompt. Schwarz (2007) described the possibility that respondents’ interpretations of questions are determined by the duration of the recall period asked. Supporting this idea, Winkielman and colleagues (1998) showed that people draw on the length of the reporting period when answering questions about themselves. When asked how often they felt angry over the past year, participants thought about rare, but intense, episodes of anger. In contrast, when the question was presented with a shorter time frame (past week), participants thought about mundane and less intense anger experiences. Anger episodes from longer recall time frames may, then, be of greater intensity than those from shorter recall periods simply because of the way the question is asked. This effect likely generalizes to the very short EMA reporting periods and to many of the phenomena that are of interest to EMA researchers.

We recommend that real-time cognitive interviewing of EMA items be conducted to evaluate whether EMA questions are understood by respondents as intended. At a minimum, we caution researchers not to assume that questions necessarily measure a construct in the same way modified from standard psychological inventories (which have lengthy or unspecified recall periods) into momentary formats. Schwarz (2007, p. 23) closed a chapter on momentary self-reporting with the admonition that we need “systematic experimental research into the cognitive and communicative processes underlying concurrent reports,” a point with which we agree. A series of targeted studies on this topic using various outcomes (e.g., continuous states and frequency) could elucidate how momentary items are interpreted.

Our second concern is that all respondents should interpret EMA questions in a consistent manner, such that the meaning of scores is directly comparable across individuals and over time. Measurement scales developed for use in traditional research settings often undergo painstaking scrutiny to ensure this consistency by conducting tests of measurement noninvariance or differential item functioning, which occurs when different groups (e.g., men versus women) interpret the content of certain items differently (Mellenberg 1982, Putnick & Bornstein 2016). In EMA settings, concerns about measurement noninvariance are amplified because items may function differentially not only between groups of individuals but also within the same individual across different situational contexts or over the course of a study. For example, a momentary fatigue item “I feel too tired to concentrate” may shift its meaning depending on whether a person is currently at work (and engaged in cognitively demanding tasks), engaged in physical exercise, or doing leisure activities (where the ability to concentrate is arguably less important). Moreover, item parameter drift can occur when the meaning of items changes over time and/or with repeated administration (Lee & Cho 2017, Liu et al. 2017). Although the necessary statistical tools are available to examine this possibility, tests of context-dependent differential item functioning and item parameter drift have rarely been applied to EMA questions. As such, we do not know if or how frequently these issues occur.

We recommend that more attention be paid to (between- and within-subject) measurement models in EMA research to understand whether group differences or context-related shifts in the meaning of momentary questions threaten valid conclusions from EMA (Schneider & Stone 2016). We note that testing these measurement models requires multiple EMA items assessing the same underlying construct. Although the use of multi-item instruments is customary in EMA research for some constructs (e.g., positive and negative affect), single-item instruments are frequently used to keep EMA surveys brief, and this practice prohibits the detection of items that function differentially. Dedicated psychometric studies may be required for this purpose, even though planned missingness designs might be a compelling alternative to reduce the number of items per survey (Arslan et al. 2021, Silvia et al. 2014).

## DO PEOPLE USE COMPARISON STANDARDS WHEN ANSWERING EMA QUESTIONS?

As is the case with most questionnaire items, we suspect that the EMA response options and the cognitive processes associated with them may shape respondents’ self-reports (Schwarz 1999). Many commonly used momentary questions employ relativistic response options such as Not at All, Somewhat, Moderately, Very Much, and Extremely (known as vague quantifiers; Wanke 2002), or they use numbers or sliding scales with similarly worded anchors. Answering such rating scale questions requires the respondent to engage in some form of relativistic comparison. The issue hinges upon how response descriptors, such as Moderately or Very Much, are compared to whom or what or when (Junghaenel et al. 2018); it seems likely that responses could be influenced by social comparisons. For example, an older person judging their level of physical symptoms relative to same-aged peers would likely give a different answer when using a comparison standard of the US population (Ubel et al. 2005). Similarly, participants could judge their momentary happiness relative to how happy they “usually” feel, or they may compare their current happiness level against their happiness experienced at the previous momentary assessments.

Controlled laboratory studies suggest that comparison standards could have a substantial impact on momentary experience reports. Watkinson et al. (2013) exposed participants to a series of standardized pressure pain stimuli varying in painfulness, and some groups had more intense stimuli than other groups (resulting in a higher average pain exposure). They found that the ratings were not in accordance with the absolute level of pain stimuli; instead, they were relative to the overall range of the presented stimuli. There was no difference in average pain ratings by group: Only the relative rankings of pain stimuli within individuals were preserved in the pain ratings—a finding consistent with range-frequency theory (Parducci & Wedell 1986). We do not yet know whether results from these laboratory studies translate to momentary ratings taken several times a day in people’s natural environments or if they extend from sensory phenomena to psychological states. It is possible, however, that momentary data collection does not eliminate biases associated with comparison standards.

Drawing on Robinson & Clore’s (2002) accessibility model, we speculate that information most readily accessible is likely to serve as a comparison standard in momentary assessment. EMA questions answered immediately before a given question may provide reference points that serve as the comparison standard for the current question. This contention could be tested by experimentally manipulating the ordering of items (e.g., presenting questions about the current social context before or after questions about mood) within EMA prompts (Schuman & Presser 1981). Another example is from EMA studies using high-frequency prompting schedules, where participants’ ratings provided on the previous prompt may still be highly accessible in memory and may serve as the comparison standard. If so, we would expect between-group (or between-person) differences to be reduced relative to within-person variation in studies with high prompt frequency. In fact, a recent meta-analysis (Podsakoff et al. 2019) suggested that the proportion of within-person variance in EMA studies increases with higher prompt frequency, and this finding could be further substantiated in studies experimentally manipulating the number of daily EMA prompts. Future work could also document the possible importance of these issues by comparing levels of experiences from momentary studies employing different types of response scales or employing the same type of scale with different anchors.

## MOVING BEYOND THE MOMENT: A REASONABLE CHOICE?

Early on, EMA researchers realized that many of the events and experiences they wished to study were either too infrequent or of such brief duration that they would be difficult to capture with randomly sampled moments focused on immediate experience. For example, infrequent arguments with a spouse are not likely to coincide with momentary assessments that occur five times a day over a 1-week EMA study. Thus, asking respondents whether they had an argument immediately before they were signaled may not provide the information required to accomplish the aims of a study.^1^

We emphasize that randomly sampled moments will by definition generate unbiased estimates of phenomena under certain circumstances, one of which is that assessment completion rates are very high (low completion rates undermine the advantages of random sampling). The logic here is analogous to the rationale for randomly sampling persons from populations with the expectation that the resulting statistics will adequately represent the population from which the sample was drawn. But the degree of error of estimates from a given sample of moments will vary depending on multiple factors. We first consider the case of an investigator wishing to characterize the average level of a variable for a group of individuals (say, argument frequency). Over a given period of time, a true frequency of arguments exists for each individual; the degree to which the observed frequency (from EMA) differs from that value represents random sampling error. Less error will occur when observed values are based on protocols with many EMA moments sampled during the study period compared with those derived from a lower density of EMA sampling (all other things being equal). Thus, there can be a reliability problem with estimates of infrequently occurring events.

Another practical issue for EMA is that sometimes researchers want to capture a summary of a state or behavior over some period prior to the prompt. Scientists investigating physical activity, for instance, may be more interested in the cumulative activity level (e.g., number of steps) over the last hour or since the last momentary assessment than in the activity exhibited at a single moment (i.e., if steps were being taken right before the person was signaled). The same may be true for pain and emotion researchers, who may be more interested in experience over the last 2 h than at a particular moment.

Finally, some researchers may wish to predict the level of a momentary state from events that may have occurred in the last few hours. For instance, a researcher might like to know if momentary perceived energy is associated with recent (say, the prior 2 h) caffeine consumption. Momentary measurement of caffeine intake (“Were you consuming caffeine right before the prompt?”) would perhaps yield very accurate data, yet unless the frequency of prompts was extremely dense (many per hour), many instances of caffeine intake might be missed. For this reason, a potentially attractive alternative is to ask about the occurrence of the state (caffeine intake) over an interval prior to the prompt.

To accommodate these needs, approaches based on EMA have been developed to capture activities over longer reporting intervals—typically over the past few hours or since the last assessment. We refer to this approach as a coverage model of EMA (cEMA; Shiffman et al. 2008) as the intention is to “cover” a longer period than the original momentary methods. One variant of this technique uses questions to inquire about events or states over a set period of time, such as the last 1 or 2 h. Another variant asks about the period of time since the prior EMA prompt (in the case of the first prompt of the day, the period would be defined as since waking). Regarding the content of what is measured, cEMA has generally asked respondents to summarize the level of an experience over the reporting period (e.g., total activity level or average anger over the period) or to report the occurrence of discrete events during the period.

We note that some studies have combined EMA and cEMA methods in a single momentary protocol—for example, asking about immediate affect with EMA and asking about recent stressful events with cEMA (Scott et al. 2017). Although efficient, such approaches raise new questions. For example, despite instructions to alter the response period (e.g., “right now” followed by “since the last beep”), do respondents persist in using one reporting period rather than alter reporting periods as instructed? To date, we are not aware of any research on this possibility.

Although of value, coverage assessments raise the possibility that recall bias and error creep into even these relatively brief assessments, especially for highly fluctuating experiences, such as affect and pain (which are likely to be more difficult to recall than more discrete behaviors or states). Perhaps informative for this discussion are investigations of how end-of-day diary data compare to momentary samples taken throughout the same day. Studies have demonstrated that recall bias consistent with peak and end heuristics can operate even within a single day’s recall of experience of pain, fatigue, and negative affect (Neubauer et al. 2020, Schneider et al. 2011), suggesting that bias can happen over a period as short as a waking day. Also relevant are studies where pain ratings were taken on a minute-by-minute basis during a short, painful medical procedure (colonoscopy) and compared with an overall rating of the period studied (Redelmeier & Kahneman 1996, Redelmeier et al. 2003). Peak and end effects were strongly associated with a summary measure of pain for the entire procedure. A detailed report described how a negative state (pain) was summarized over brief periods (up to 40 s) (Ariely 1998), where the intensity of a heat stimulus (to induce pain) was carefully manipulated to create patterns of the stimulus over the experimental period (participants were sequentially exposed to many pain intensity patterns). Recalled pain ratings were not simple summations of pain levels during the periods: The trend in pain intensity over the period (decreasing or increasing) and the pain level at the end of the exposure period both influenced retrospective recall. These studies indicate that the processes underlying recall in cEMA (at least of continuous states) may be quite complex.

Although the above arguments seem particularly germane to continuous states, the cEMA method is also used for assessing the presence/absence or frequency of discrete events (Shiffman et al. 2008), such as particular interpersonal occurrences, well-defined symptoms, and psychological stressors. We think that these phenomena are less susceptible than continuous states to retrospective distortion because the memory demands of this task appear less onerous (Himmelstein et al. 2019). Nevertheless, it is plausible that recognized distortions in event reporting (e.g., forward telescoping; Schwarz & Oyserman 2001) could operate even with short recall periods.

Despite widespread use of cEMA, we do not know of any systematic research verifying that the coverage technique is working as intended, including the basic question about whether respondents are using the requested reporting time frame. As with momentary assessments, one approach to explore these issues would be to examine how respondents report experiences and behaviors over the specified coverage periods. The first avenue of research we suggest is again cognitive interviews. A study mentioned above (Wen et al. 2021) on EMA recall periods included an experimental arm testing a reporting period used in cEMA. The version of cEMA was “since the last prompt,” and the study employed the real-time telephone interview protocol described above. To our dismay, the protocol for our cognitive interviewing protocol was not sufficient for fully exploring the complexity of responses to our question about how EMA questions were answered. Hence, we were unable to reliably code the responses and understand how cEMA reporting periods were used by respondents.

Although the first effort failed, we believe that the cognitive interviewing method could enlighten us about the coverage model, but considerable preparatory work developing the interview (e.g., delineating the sorts of responses that could be expected when respondents are discussing how they answered questions, and training interviewers about them) will be necessary for a successful study. Even with this preparation, there are likely to be challenges. For example, how are brief flares of emotion during the cEMA reporting period summarized? How does intensity vary over the period, and how is it summarized?

A second approach might use a variant of the Ariely pain study design (Ariely 1998), expanding the reporting period to 2–3 h to be consistent with the periods typically used with cEMA. This could be an attractive alternative to cognitive interviewing because the investigator experimentally tests the impact of various topographies of stimuli that are meant to elicit emotions, pain, and other sensory states and then determines how features of these stimuli influence recollection. Furthermore, it would be possible to investigate if there were particular techniques to reduce potential recall bias in cEMA in participants’ reports. For example, drawing on procedures used in the Day Reconstruction Method (Christodoulou et al. 2014, Kahneman et al. 2004), asking the participants to systematically review or relive the experiences of the past few hours in their minds could improve recall accuracy.

Although cEMA targets a greater proportion of the day, EMA methods will still have limited effectiveness at capturing very infrequent events, such as those that occur once a month or even less frequently. Most major life events fall into this category. Executing EMA protocols over weeks or months is either inefficient or simply infeasible. A compromise solution has been developed: so-called EMA burst designs (Sliwinski 2008), which employ multiple periods of EMA data collection. A variant of this methodology is combining EMA bursts with ongoing monitoring of participants so that the occurrence of an infrequent event triggers a burst of measurement proximal to the event.^2^

## HOW IMPORTANT IS TRAINING PARTICIPANTS FOR EMA?

We may optimistically assume that participants are adequately instructed prior to engaging in an assessment task, especially one as complex as EMA monitoring, yet information on this important aspect of EMA study implementation is limited in the literature. With the rapid development of new technologies, including smartphone applications and novel wearable devices often incorporated in EMA studies, it is possible that participants may not be able to intuit appropriate use in the absence of explicit training.

Many EMA studies do instruct participants in some manner, but evidence documenting the presence of training in EMA studies is scarce. Some meta-analytic studies have found encouraging rates (e.g., 69% of studies examining substance use explicitly reported training participants; Jones et al. 2019), whereas others have found less encouraging rates (e.g., 25% of studies studying physical activity; Degroote et al. 2020). It is difficult to ascertain whether such variability reflects differences in the target samples, domain of research, reporting standards, or other factors; we suspect that some, at least rudimentary, training is conducted in most studies, but it may not always be reported. More information about the occurrence of training needs to be routinely provided, and when it does, more details need to be reported about the nature, extent, timing, and other aspects of training content and delivery (e.g., Kirtley et al. 2021, Smith et al. 2019).

Even when training does occur, there is considerable variability in aspects of training content and delivery. Although many researchers assert that instruction may lead to improved data quality (e.g., fewer missing data, more timely responses to signaled reports, more accurate use of rating scales, greater participant retention; Robinson et al. 2007, Trull & Ebner-Priemer 2020), there is scant evidence available to evaluate such claims.

At one end of contemporary practice, participants are trained in the laboratory or in the field with face-to-face interactions. Such training may include specific practice with the data collection device—for instance, how to interact with the device itself, demonstration of the survey program, and completion of several practice assessments (often supplemented with diagrams and pictures). Additionally, some studies implement formal run-in periods (e.g., Clauw et al. 2008, Scott et al. 2015) wherein participants complete momentary reports for several days and only those participants demonstrating good compliance are retained for ongoing research (a process that results in presumably higher compliance rates but also raises issues about selection biases and resultant generalizability concerns). Generally, we posit that having the ability to ask questions about the study procedure, practicing that procedure in situ, providing an explicit context about when to respond to prompts, standardizing the terminology (e.g., what constitutes a stressor), and having research personnel provide feedback about participants’ practice performance should improve adherence to EMA study protocols and may enhance data quality. Other aspects of study quality, such as participant satisfaction and retention, may be enhanced as well (Kost et al. 2011).

At the other end of the continuum, studies may not include hands-on training or have any practice with the data collection device or application whatsoever. At times this may be due to perceived burden related to the length of an initial visit or situations of remote administration, as in the case of Internet-based studies where there is no direct contact with participants. Although we understand the pragmatic considerations on this point, there are methods that can efficiently and/or remotely provide some training in most cases (e.g., training documents/FAQs provided, phone training sessions, training videos). Some evidence suggests that training participants in the use of rating scales in daily diary studies can reduce missing data and improve the internal consistency of diary ratings (Smith et al. 2016).

To our knowledge, there have not been any studies investigating the effects of training (e.g., training type, duration of training) on participant retention, compliance rates, and data quality that are likely to affect a study’s internal and external validity specifically in EMA studies. Similarly, even though systematic reviews have attempted to quantify the nature and extent of training parameters in EMA studies (Heron et al. 2017) and training is typically noted as an important consideration (e.g., Trull & Ebner-Priemer 2020), few reviews have related descriptive information about training parameters to indicators of data quality. Without such a systematic review or high-quality studies on the topic, it is impossible to recommend best practices, although one may err on the conservative side and suggest the inclusion of the most comprehensive training that is pragmatically feasible.

Another broad approach to examining these issues is to experimentally manipulate training parameters. As noted above, we have conducted work using this approach, contrasting participants randomly assigned to receive relatively rudimentary versus more comprehensive and detailed training with regard to observed fidelity of respondents’ use of the intended recording period (Wen et al. 2021). In short, providing more extensive training greatly improved the proportion of respondents reporting on the desired time period when prompted compared with another group who received minimal training. We believe this is some of the first experimental evidence that training is an important ingredient for successful EMA studies.

We recommend additional studies to evaluate the impact that thorough training has on study processes and data quality to inform future EMA studies. This research should address basic questions including the following: Is training a necessary component for optimal EMA data collection? If so, what aspects of training (e.g., the amount, content, and nature of training) are important and for what types of study-related process and quality indicators? Additional research is needed to examine the boundary conditions of such effects (e.g., is there evidence of moderation by sample characteristics, or do training effects emerge in particular responding contexts, such as distracting environments or complex items?).

## PARTICIPANT SELECTION BIAS IN EMA STUDIES

Selection related to who participates in EMA studies is important for interpreting the findings (e.g., Scollon et al. 2009). EMA studies are characterized by significant participant burden, and this feature likely impacts who is willing to engage in these studies. It is first necessary to explain what we mean by participant burden, which we define broadly. A component of burden is the amount of time required to complete the prompts required by an EMA protocol, which may be computed by taking the average amount of time to complete a momentary assessment multiplied by the number of prompts per day, which is in turn multiplied by the number of study days. For example, the amount of assessment time for a study of 14 days with five prompts per day and each prompt requiring 2 min is 140 min. Time could be added for training (30–45 min) and study debriefing (5–10 min), yielding a total of about 3 h. A second component of burden is the interruptions associated with completing several daily prompts per day; this undoubtedly varies widely depending upon a participant’s occupation and circumstances. People with active schedules might have difficulty responding to prompts, and interruptions could be particularly disrupting in some circumstances (e.g., during important work conversations or activities).

Given the burden associated with EMA studies, individuals who agree to participate in these studies are likely to differ in many ways from those who do not: They may have relatively high levels of motivation, interest, and perceived ability to complete the required reporting tasks. This may skew EMA participant samples toward individuals who find meaning in their participation (e.g., patients hoping the research will help others with the same illness), those who are more familiar with electronic devices (younger people and those who are computer savvy; Keusch et al. 2019), those with fewer professional and/or personal demands, and/or those with certain personality characteristics (e.g., high conscientiousness, openness to experience; Cheng et al. 2020). The magnitude and direction of such selection effects on the associations being investigated will typically not be known at the outset of a study and pose a threat to the generalizability and external validity of findings.

Achieving a deeper understanding of participant selection biases in EMA studies will not be simple; in fact, there probably is not a single answer to the question because answers are likely to depend on a host of factors concerning the purpose and design of studies. Motivational issues may be less salient in selection bias in medical studies, where patients could imagine themselves deriving benefit from the research, than in studies of “daily life” (a common way to label momentary studies), where benefits are not apparent. Formal design factors that are presumably associated with perceived burden, such as survey length or number of recording days, are also likely to impact who is willing to participate (Smyth et al. 2021).

Despite these difficulties, we believe it is important to understand selection effects because having some sense of the magnitude of the problem could facilitate interpretation of findings. One straightforward suggestion is that researchers refine recruitment methods to allow for comparisons of information about those who agree to participate in momentary studies with information about those who decline such invitations. Common wide-ranging methods for recruiting EMA participants, such as radio advertisements, widely posted flyers, and social media, are not particularly helpful in this regard; in fact, one does not even know the rate of participation as there are no data on how many individuals hear or view the advertisements. In contrast, studies of consecutive patients in a hospital clinic who are offered participation in a momentary study, where basic information is likely to be available about all admissions, could allow for comparisons of at least basic demographic variables (Benedict et al. 2019). However, simple demographic variables may not be the most useful for predicting participation rates (Keusch et al. 2019); as mentioned earlier, confidence in using a smartphone, for example, may influence one’s interest in volunteering for an EMA study.

Alternatively, studies could be designed to examine participant uptake rates using sampling techniques with information available about those invited to participate (such information might be available from specialized survey or marketing firms). In this manner, all of those invited to the study could be compared with those who ultimately completed it. Furthermore, for individuals who opened the invitation (e.g., an email or written introductory letter), efforts could be made to collect additional information about them. For instance, respondents could answer a brief questionnaire about their interest in the study and why they made the decision to participate or not. Completion of this questionnaire could also be incentivized and made extremely easy to complete and return (e.g., with several response modalities such as email, snail mail, and telephone). Although only dealing with a subset of the full group to whom the study was offered, the results would nevertheless be informative regarding who participates in momentary studies.

## MISSING EMA ASSESSMENTS: IMPACT OF MOMENT SELECTION BIAS

For good reasons, the topic of missing EMA responses is prominent in researchers’ minds. Missing EMA data lead to a loss of information, decreased statistical power, potentially biased parameter estimates, and weakened generalizability of findings (Schafer & Graham 2002); this issue has also been termed “compliance bias” (van Berkel et al. 2020). Dealing with missing responses is especially complex in EMA studies because of their intensive longitudinal nature. This is evident in each of the topics discussed below: how compliance rates are reported and ascertained, how missing momentary assessments affect the data and results, and how to understand potential reasons underlying missing assessments.

The majority of momentary studies report at least basic compliance information. For example, systematic reviews find that 65% of ambulatory assessment studies in psychopathology research report on their definitions of compliance (Trull & Ebner-Priemer 2020) and that a similar two-thirds of studies on chronic pain document average rates of missed EMA prompts (May et al. 2018). When reported, EMA compliance (typically defined as the percentage of beeps responded to of the total number requested/prompted) ranges between 70% and 85% ( Jones et al. 2019, May et al. 2018, Wen et al. 2017). However, there is considerable variability in the specific information that is reported and in how compliance rates are computed. In itself, this variability is concerning because it can obscure understanding of a study.

Given that each EMA study participant has their own compliance rate, documenting compliance as a single summary measure (e.g., for the average participant) can conceal important information. Imagine a study where several participants complete very few assessments and compliance rates are computed only on those with higher completion rates (e.g., the sample used for analyses). Imagine another study with the same completion rates but in which all respondents are used to compute compliance statistics. The latter study will appear to have worse compliance than the first. Inconsistencies like these can be overcome by reporting compliance rates for the full and analysis samples together with distributional information (e.g., range, percentages in different compliance rate brackets). We support efforts to enhance compliance reporting. Standards for reporting compliance have been presented (Stone & Shiffman 2002, Trull & Ebner-Priemer 2020), and a checklist for reporting EMA studies has also been proposed (Liao et al. 2016).

With this background in mind, we now turn to two broad concerns stemming from poor compliance with EMA protocols. The first is how missing assessments can affect the interpretation of study results; the second concerns reasons for missing assessments.

### Effect on Interpretation of Results

Rubin’s (1976) seminal work classified missing data into three distinct categories. According to Rubin, data are missing completely at random (MCAR) if their missingness is unrelated to any measured variables, such as prompt responses that were not recorded because of random technical difficulties. This type of missing data reduces statistical power but otherwise does not compromise study results. The second category describes data that are missing at random (MAR). Contrary to what the name might suggest, MAR actually implies that data are systematically missing, which can severely bias study results. However, MAR means that which values are missing can be attributed entirely to the values of other variables that are not missing. For example, suppose that momentary physical activity reports (the outcome variable) were more likely missing when individuals were at work or in the evening (when physical activity levels are lower). These data would be MAR, provided that missingness was unrelated to physical activity, once momentary location (e.g., work, home) and time of day were accounted for. Finally, data are missing not at random (MNAR) if the likelihood that a value is missing depends upon the (partially unseen) values of the variable that has the missing information. In the physical activity example, MNAR would occur if EMA prompts were more likely skipped when individuals were currently exercising.

In the past two decades, there has been a dramatic shift away from ad hoc techniques (e.g., deletion methods) that assume that data are MCAR. Contemporary multilevel analyses used in EMA research commonly assume an MAR mechanism accommodated by maximum likelihood parameter estimation or (less frequently used in EMA research) multiple imputation procedures (Schafer & Graham 2002). Additionally, techniques to accommodate MNAR (most prominently, pattern-mixture models and selection models) have proved useful in traditional longitudinal contexts (Enders 2011), and extensions have recently been proposed for multilevel analyses of EMA data (Cursio et al. 2019, Lin et al. 2018). These are important developments even though considering an MNAR mechanism requires that the statistical analysis must incorporate a submodel describing the propensity for missing data, which can be very challenging to implement in EMA studies characterized by many repeated observations and many resulting missing data patterns. MNAR analyses also rely heavily on untestable assumptions (e.g., normally distributed latent variables). A common viewpoint is that MNAR analyses should serve not as a routine replacement of analyses that assume MAR but rather to help explore the sensitivity of results to different assumptions (Enders 2011).

Despite the virtues of maximum likelihood estimation to accommodate MAR, there are noteworthy pitfalls that EMA researchers may not always be aware of. One important concern is that selection biases under MAR are eliminated only if the missing values can be attributed entirely to other variables that are themselves observed. In EMA research, the best candidate variables to explain why a variable is missing are often other EMA questions from the same prompt, but these are also missing whenever a participant skips a prompt. To enhance the plausibility of MAR, it may be beneficial to focus attention on variables that are still observed when EMA prompts are missing; examples include temporal variables (time of day, day of week) and passively recorded ambulatory assessments (e.g., geo-location, accelerometry).

Additionally, even if the “causes” of missing EMA data are recorded, the MAR assumption only holds if these variables are actually incorporated in the analysis. In fact, statisticians have argued for an inclusive strategy whereby one includes many variables that could explain the missingness (Collins et al. 2001). Implementing this recommendation, however, poses real challenges for EMA researchers because adding many variables as covariates alters the substantive research question merely to accommodate missing data. This problem can be circumvented using multiple imputation techniques because observed variables that are used to impute missing values do not enter the analysis model directly. To date, multiple imputation is rarely applied to multilevel EMA data, in part because different analysis models (e.g., models with random intercepts, random slopes, and cross-level interactions) can require specifically tailored imputation strategies (Enders et al. 2020, Grund et al. 2018). Nevertheless, embracing these techniques in EMA research could greatly benefit the field by reducing biases associated with missing data.

It should be clear that studies with high compliance rates are less susceptible to these selection biases, yet definitions of a high level of compliance are open for discussion. In non-EMA (often single-assessment-point) contexts, Schafer (1999) maintained that missing data rates of 5% or less are inconsequential, and Bennett (2001) argued that results are likely biased when more than 10% are missing. Our impression is that many EMA researchers believe a level of 75% within-person compliance is adequate and 90% is excellent. This evaluative judgment is difficult to make given the complexities of how selection factors operate to generate bias. With this in mind, it seems prudent for EMA research to increase efforts to understand the reasons for missing assessments because doing so would help researchers (*a*) devise strategies to reduce missing data and (*b*) evaluate selection biases that are prevalent in EMA studies.

### Reasons for Missing Assessments

There are many reasons for missing assessments. Technological failures of the data collection apparatus can result in missing EMA data. A particularly vexing problem for some smartphone-based apps is that software updates can interfere with the operation of the app, potentially resulting in lost data. For systems that rely on server-based administration, interruption of cell or Wi-Fi service can disrupt data collection and/or transmission. How should technological failures be considered in terms of selection bias? It is unlikely that software updates are systematically related to outcomes, and resulting missingness may often be considered MCAR. However, access to cell service and Wi-Fi could depend on location, which in turn may be associated with factors related to selection bias (e.g., service is more limited in remote areas where social interaction is less likely).

Missing assessments based on situational factors are more likely to contribute to selection bias because, in many cases, the situational factor will be related to the outcome variable. Biased sampling of momentary affect, for example, could occur if participants are not willing to answer prompts when they are in the midst of a marital dispute or engaged in other activities that discourage responding, such as participatory athletics. These are all examples where the missingness mechanism is likely MAR. Other situational factors include those based on the level of outcome variables themselves, such that the missingness is MNAR; mood and pain are cases in point. Respondents who are experiencing high pain when prompted may be disinclined to answer given the pain; this could have profound impacts on estimates of average pain levels and on estimated relationships between pain and other variables. Studies that have examined situational predictors of noncompliance suggest that engagement in behaviors that draw attention away from participation (e.g., drinking alcohol, exercising) lowers momentary compliance (McLean et al. 2017, Rintala et al. 2020, Silvia et al. 2013, Sokolovsky et al. 2014). There is little evidence that momentary experiences (e.g., mood, stress) affect compliance. Yet, as mentioned above, momentary experience data are always missing when prompts have been missed, such that this evidence is largely limited to experiences reported at previous (nonmissing) prompts (Dzubur et al. 2018, Messiah et al. 2011, Schüz et al. 2013).

Situational factors are also likely to contribute to response delays, a subtle yet potentially important form of moment self-selection bias. When participants are prompted in unexpected or inconvenient situations, they may not respond immediately but instead delay (i.e., self-select the response timing) by several minutes. A handful of studies have compared EMA ratings across immediate and delayed responses and have reached mixed conclusions (Affleck et al. 1998, Eisele et al. 2021b). These studies are difficult to interpret with confidence, however, in the absence of ground truth. For example, immediate and delayed responses may look similar (e.g., low pain ratings in both cases) precisely because the delay disguises actual differences (e.g., the participant was in pain when prompted and waited until the pain subsided).

Participants’ motivation to complete the assessments may also affect compliance and response delays. One source of motivation long recognized by researchers is participant compensation, but evidence supporting the effectiveness of financial incentives to promote adherence in clinical research is mixed (Giles et al. 2014). Another factor that relates to motivation is participant burden. Evidence suggests that long EMA surveys (e.g., 60 compared with 30 items) may reduce compliance (Morren et al. 2009), whereas more frequent EMA prompts have not been found to affect compliance rates (Eisele et al. 2022) and may even be related to higher compliance in some samples (Wen et al. 2017).

We see tremendous value in better characterization of moment selection biases. A researcher can make a thoughtful decision about how to treat missingness if they know why a prompt was missed. One avenue for such investigations could use interviews or open-ended queries following some period of momentary recording (e.g., at the end of the day) to ask why the reports were missed (Aaron et al. 2004). Another avenue is to use microrandomized trials (Li et al. 2020); participants could be sequentially randomized to different conditions that are thought to affect compliance (e.g., reward messages for completing surveys, prompts tailored in time or to specific locations) to experimentally study the impact of these conditions on compliance rates.

## ARE EMA MEASUREMENTS RELIABLE?

Reliability of measurement is a core psychometric feature, yet reliability estimates are not commonly reported in applied EMA research, and they have been largely confined to psychometric investigations (Carlozzi et al. 2018, Edmondson et al. 2013, Eisele et al. 2021a, Murray et al. 2022, Scott et al. 2020, Versluis et al. 2021). This is unfortunate because unreliability due to measurement error has many undesirable consequences including loss of statistical power (Snijders 2005) and potentially biased parameter estimates (Muthén 1997). Reliabilities in momentary studies depend on the research question at hand and on how the scores are used in the analysis. Broadly speaking, we can distinguish errors in person-level measures derived from EMA data and errors in the measurement and comparison of different moments within a person. We note that this broad distinction brushes over many nuanced reliability considerations relevant in EMA studies (Nezlek 2011).

At the person level, one major source of unreliability is sampling error. It occurs because EMA studies sample only a limited number of observations from each person. Whenever EMA scores are aggregated across measurement occasions, the resulting summary scores contain sampling error. A classic example is when EMA researchers calculate a person’s mean on a variable—for example, to distinguish individuals based on their mean affect levels. More recent work has developed summary measures that distinguish individuals on dynamic aspects of experience, including how much individuals differ in within-person variability (e.g., affect instability) or in temporal dependencies of their experiences (e.g., affect inertia) (Ram & Gerstorf 2009).

Simulation studies suggest that person-level measures derived from EMA vary dramatically in reliability unless a large number of measurement occasions is available to reduce the sampling error (Du & Wang 2018, Estabrook et al. 2012). Such results cast doubt on the usefulness of some person-level measures of experience dynamics, and their reliability requires rigorous testing in empirical samples. For individual differences in means, intraclass correlation coefficients (ICCs, often with adjustment using the Spearman–Brown prediction formula) are an indicator of reliability. A systematic review found that less than 10% of articles reported ICCs or multilevel variance components needed to calculate them (May et al. 2018). Calculating reliabilities of dynamic person-level measures is more complex but could be accomplished by employing parallel forms of EMA measures (Eid & Diener 1999) or by deriving parallel indices from odd and even sampling occasions (Wendt et al. 2020).

We now turn to the second type of measurement error that occurs when the goal is to investigate moment-to-moment changes (and/or day-to-day changes) within individuals. The challenge is to distinguish “true” within-person changes from momentary (occasion-specific) measurement error. Statistical procedures using generalizability theory (Cranford et al. 2006) and multilevel factor analysis (Geldhof et al. 2014, Lai 2021) have been developed to estimate within-person reliabilities pooled over respondents. Techniques have also been proposed to estimate within-person reliabilities that are individual-specific (Hu et al. 2016, Schuurman & Hamaker 2019). These are important developments because it may not be realistic to assume that all individuals are measured with the same reliability (see Fisher et al. 2018). Just as individuals can differ in their within-person relationships among variables (which is often an essential part of EMA data analysis), they can differ in the within-person reliability of each variable measured.

In summary, the complex nature of EMA data and continuously emerging new perspectives for how to measure and quantify the ebb and flow of individuals’ experiences create many challenges for researchers who wish to estimate and document the reliability of EMA measurements and use this information to inform their findings. Ongoing developments for improved reliability estimation need to be made available and easily accessible to be implemented in applied settings. As reliabilities are always sample dependent, we encourage researchers to document relevant reliability coefficients in all empirical EMA studies. Not only will this enhance transparency about the quality of conclusions from EMA studies but it will also provide the necessary information for subsequent research to make informed study design decisions.

## WHEN SHOULD EMA BE CONSIDERED A GOLD STANDARD?

Despite the concerns mentioned herein, momentary assessment is often accepted as a gold standard for measuring experience, just as ambulatory blood pressure monitoring is a gold standard for blood pressure in the natural environment. There are many considerations to discuss in taking this position. Scollon and colleagues (2009) remind us that momentary self-reports are likely sensitive to biases that have nothing to do with high accessibility to the information, such as the impact of social desirability and cultural norms (also see Schwarz 1999). A general perspective on self-reports (Tourangeau 1984) breaks self-reporting tasks into the following components: understanding the intent of the question, retrieving relevant information for answering the question, summarizing the information accessed, and selecting an appropriate response option. EMA clearly reduces recall bias given its focus on the current moment, but the three other components of the model are areas where momentary methods face the same difficulties as other survey methods.

Careless responding is another aspect of self-reporting that is applicable to EMA and may threaten measurement validity. Inattentive or careless responding is a form of “satisficing” (Krosnick 1991); it occurs when respondents complete survey assessments but do not expend the mental effort required to read and interpret the questions or to generate meaningful answers. This hidden form of noncompliance has been well documented in traditional (Internet-based) survey research settings, where about 7–15% of respondents are characterized as inattentive (Jaso et al. 2022, Meade & Craig 2012, Schneider et al. 2018). We know little about the prevalence or implications of careless responding in momentary data collection (Eisele et al. 2022). If it is substantial, then we need to explore the reasons. It might be related to completing the same set of questions many times a day for many days, which might result in mental disengagement from the task or in situations where respondents are highly engaged in something when signaled, resulting in less attention being paid to the momentary assessment.

Another manifestation of the gold standard debate is the apparent competition between EMA and retrospective assessment for measuring particular constructs, with the implicit question at hand being “Which is most valid?” Studies have shown that recall of pain is higher than the average of many momentary reports over the same time period (Broderick et al. 2006, Jensen et al. 2008, Miron-Shatz et al. 2009, Stone et al. 2004, van den Brink et al. 2001) and that there is a substantial association between the two types of reporting (Broderick et al. 2008, Kikuchi et al. 2006). If one takes the position that momentary reports are valid (in the sense that they are measuring what we want them to measure), that the sampling of moments has been random, and that protocol compliance was high (eliminating selection effects on moments due to nonrandom sampling), then one could reasonably argue for the use of a momentary average. Supporting this argument, one could muster many references showing that cognitive heuristics and memory failures result in faulty retrospection, and thus recall would be less preferable than the averaged momentary reports.

So, should we always choose the momentary summary? Hardly. There is clear evidence regarding the prediction of some future pain-related behaviors that broader/global remembrances of pain over a period of time—however constructed from momentary experiences—are better predictors of certain behaviors than summaries of momentary experience. Kahneman and colleagues have referred to this distinction as experienced utility (momentary reports) versus decision utility (retrospective reports) (Kahneman et al. 1997, Redelmeier & Kahneman 1996).

The takeaway point in this example is that both momentary and recall measures are addressing the construct of pain intensity, yet they appear to be assessing somewhat distinct aspects of the construct (Conner & Barrett 2012). This important conceptual distinction affects the choice of assessment approach for a given research question. For pain intensity in our current example, the following arguments can be advanced. Momentary measures may be more relevant for studying physiological processes given the research showing that qualities of the immediate environment (including time of day) are associated with, for instance, hormone production (Smyth et al. 1998, Stone et al. 2001). Thus, momentary assessments may be preferable for this purpose given the inability of recall measures to predict such fluctuations and their tendency to focus on particular aspects of daily experiences (such as peak experiences). Conversely, a recall pain intensity measure may be closer to the construct desired if one aims to predict later health behaviors.

The gold standard designation is, then, dependent upon the precise conceptualization one has of a construct. In this regard, EMA does not by default have a superior status conferred upon it. As both momentary summaries and recall assessments may be predictive of important outcomes, we must be explicit about the theoretical framework we adopt in a particular study and show how the measures (momentary or recall) are consistent with the framework. We believe that more attention to this issue and additional empirical support for these distinctions are warranted.

## FUTURE DIRECTIONS

We present suggestions and recommendations for nine topics related to advancing the field of momentary assessment. To understand whether participants are reporting their momentary experiences, we recommend real-time cognitive interviews of EMA participants in the field to acquire information about how questions are answered. To better characterize how respondents interpret the information sought in momentary questions, we recommend two approaches (cognitive interviewing and the application of measurement models) to detect context-related shifts in question meaning. Comparison standards are a potential source of extraneous variance in momentary reporting, and we suggest novel research to examine comparison standards and range of response effects. Some EMA studies extend the reporting period from a moment to a longer (e.g., hours) period; we recommend that studies investigate the possibility that heuristic and summary processes differentially influence reports from extended recall periods in EMA. Momentary recording likely benefits from comprehensive training of participants, although little evidence supports this claim. We recommend experimental studies of training protocols that differ by comprehensiveness and modality to provide initial best practice recommendations. Momentary studies can be burdensome, and only some individuals may have an interest in participating. We describe the need for studies that allow computation of true uptake rates and can detect differences in those who do and do not participate in EMA studies. Missing data can be a major problem for momentary studies; we recommend collecting additional data about the reasons for missingness and associated biases and applying advanced statistical treatments of missing EMA data. Reliability of measurement is essential for successful EMA research, and we discuss issues that have impeded clarity on this point. One suggestion is that the reliabilities of measures to capture dynamic, within-subject processes should receive special attention given that early indications suggest inadequate reliability of some EMA measures. Finally, we suggest careful consideration of a study’s conceptual basis to aid decisions about the most appropriate study design and prior to selecting momentary data capture.

## CONCLUDING REMARKS

Our goal for this review was to raise and discuss issues regarding the collection, use, and interpretation of momentary data that we feel have not received enough attention yet may be of considerable import for the field of momentary data capture. In doing so, our intent is not to question the overall value of the methods—they certainly have advanced understanding in many fields in important ways, and investigators have been able to delve into the dynamics of experiential phenomena in ways that were previously not possible—nor is it meant to discourage researchers from applying the methods in their own studies. Rather, we have tried to stimulate a lively exchange about issues that have not, at least to our thinking, been fully discussed and resolved. New research addressing these issues will ultimately be beneficial for advancing the science of EMA by enhancing the performance of the techniques, strengthening the psychometric properties of the resulting data, and facilitating appropriate understanding and interpretation of EMA study results.

## SUMMARY POINTS

1. Ecological Momentary Assessment (EMA) has become a standard data capture tool in the behavioral and medical sciences, yet there are several issues that require attention for its potential to be fully realized. The following topics are covered in this review.
2. When instructed to make momentary assessments about their experiences, do participants actually report their current experiences, or do they answer questions with other, out-of-protocol time frames?
3. Are respondents interpreting the content of the questions asked in momentary assessments as intended?
4. When answering momentary questions, do participants use comparison standards for formulating their answers, and how might this affect their responses?
5. Is it reasonable to extend the assessment time period from the moment before the prompt (typical EMA) to longer periods of up to several hours (coverage EMA)?
6. Is training participants how to make momentary assessments important for achieving optimal data?
7. How likely and important is participant selection bias in EMA studies?
8. What is the impact of missing momentary assessments for the interpretation of EMA data?
9. What are considerations for evaluating the reliability of measurements in EMA?
10. When should momentary assessments be considered the gold standard for measuring a construct?

## Footnotes

1 We note that other EMA sampling schedules (e.g., event-driven assessments) or schedules that incorporate denser sampling at times when events are more likely to occur could be considered for capturing infrequent events (Himmelstein et al. 2019, Shiffman et al. 2008).

2 We are currently using this method in the Understanding America Study at the University of Southern California (https://uasdata.usc.edu/index.php); in addition to triggered bursts, the protocol also collects bursts taken at random time points for comparison.
